# Comparative Demography of an At-Risk African Elephant Population

**DOI:** 10.1371/journal.pone.0053726

**Published:** 2013-01-16

**Authors:** George Wittemyer, David Daballen, Iain Douglas-Hamilton

**Affiliations:** 1 Department of Fish, Wildlife and Conservation Biology, Colorado State University, Fort Collins, Colorado, United States of America; 2 Graduate Degree Program in Ecology, Colorado State University, Fort Collins, Colorado, United States of America; 3 Save The Elephants, Nairobi, Kenya; 4 Department of Zoology, University of Oxford, Oxford, United Kingdom; Australian Wildlife Conservancy, Australia

## Abstract

Knowledge of population processes across various ecological and management settings offers important insights for species conservation and life history. In regard to its ecological role, charisma and threats from human impacts, African elephants are of high conservation concern and, as a result, are the focus of numerous studies across various contexts. Here, demographic data from an individually based study of 934 African elephants in Samburu, Kenya were summarized, providing detailed inspection of the population processes experienced by the population over a fourteen year period (including the repercussions of recent increases in illegal killing). These data were compared with those from populations inhabiting a spectrum of xeric to mesic ecosystems with variable human impacts. In relation to variability in climate and human impacts (causing up to 50% of recorded deaths among adults), annual mortality in Samburu fluctuated between 1 and 14% and, unrelatedly, natality between 2 and 14% driving annual population increases and decreases. Survivorship in Samburu was significantly lower than other populations with age-specific data even during periods of low illegal killing by humans, resulting in relatively low life expectancy of males (18.9 years) and females (21.8 years). Fecundity (primiparous age and inter-calf interval) were similar to those reported in other human impacted or recovering populations, and significantly greater than that of comparable stable populations. This suggests reproductive effort of African savanna elephants increases in relation to increased mortality (and resulting ecological ramifications) as predicted by life history theory. Further comparison across populations indicated that elongated inter-calf intervals and older ages of reproductive onset were related to age structure and density, and likely influenced by ecological conditions. This study provides detailed empirical data on elephant population dynamics strongly influenced by human impacts (laying the foundation for modeling approaches), supporting predictions of evolutionary theory regarding demographic responses to ecological processes.

## Introduction

Detailed demographic data are available for only a handful of wildlife species of high economic or conservation value [1]. For the limited number of species that have been studied, data from multiple populations are typically not available, with existing information usually compiled from a single population [2]. As such, we often lack information on life history strategies across species and ecological systems, limiting our capacity to assess demographic responses to human pressure and ecological changes [3], [4]. Understanding species specific responses is of particular interest for threatened species or those that are economically salient to their range states.

One of the better studied tropical, large ungulates is the African elephant, for which multiple studies using individual based monitoring or culling data provide demographic parameters. The African elephant plays a keystone role [5] in the diversity of habitats it occupies (from deserts to rainforests) by influencing canopy cover [6], affecting species distribution [7] and dispersing seeds [8]. The state of elephant populations thus is critical to the integrity of the ecosystems it inhabits [9]. Further, this species is of high economic value in terms of commercial trade (for ivory) and tourism as well as being a flagship species. The conservation status of African elephant populations vary broadly across the continent with recent eradications in parts of Central and West Africa [10] and nearly a quarter of the species inhabiting one region in Botswana [11]. As such, its designated conservation status, trade in its products [12] and management of the species [13] are contentious [14]. As a species of high conservation concern and extreme life history strategy, with the longest mammalian gestation period (as well as long reproductive life), understanding the demographic status and response to different human pressures and ecological conditions is invaluable for theoretical and practical applications. Most of the existing data on elephants, however, were compiled from well protected populations with stable or increasing populations at the time of the study. Unfortunately, these data do not represent the status of many elephant populations across the continent [10], [11], [15], and it is imperative conservation and management bodies have comparative data on populations experiencing greater pressure to determine differences in population status and response to human impacts.

Here we summarize the demographic data derived from 14 years of continuous, individual based monitoring of the Samburu elephant population in northern Kenya [16]. This area is of high conservation interest as one of the Convention on International Trade in Endangered Species (CITES) Monitoring of Illegal Killing of Elephants (MIKE) sites, a program to assess the relationship between legal ivory trade and illegal killing of elephants [17], [18]. During the study period, this population was subject to consistent human pressure and predation [19], [20] the impact of which recently spiked [21]. Life table metrics from the Samburu population are presented and its demography during periods of relatively low and high human induced mortality compared and contrasted with information from published demographic studies of African elephants in different ecosystems. The demographic processes related to varying degrees of human pressure and different ecological zones are discussed in relation to the conservation status of this species.

## Methods

### Study System

**Figure 1:**
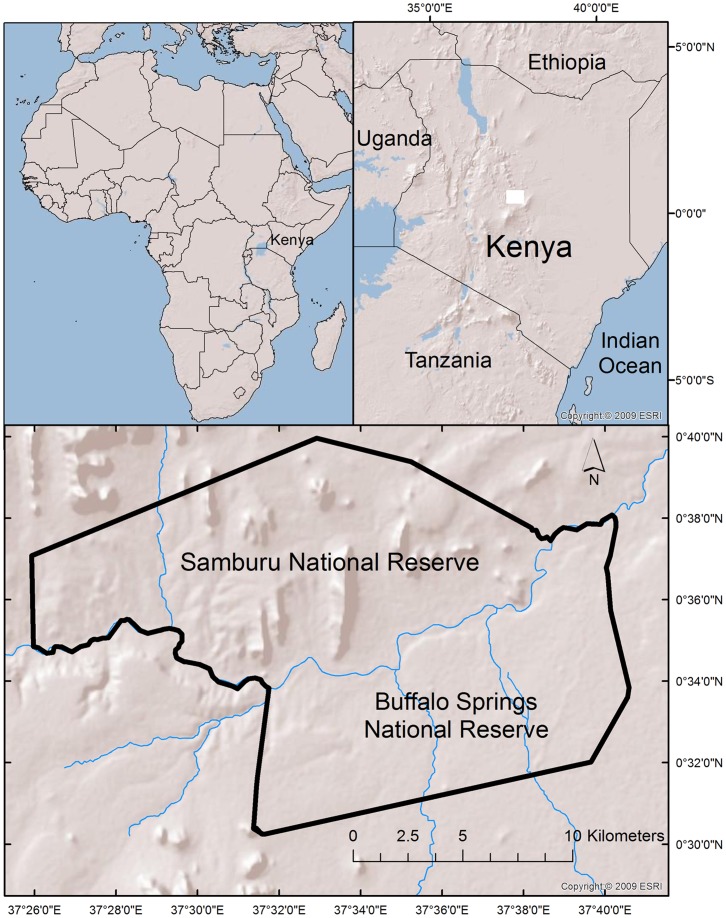
Map of the Samburu and Buffalo Springs National Reserves in Kenya, East Africa.

Individually based demographic data on the Samburu elephant population were collected through an individual identification study of all elephants inhabiting the semiarid, 220 km^2^ Samburu and Buffalo Springs national reserves in northern Kenya (0.3–0.8° N, 37–38°E) (Fig. 1). These reserves are centered on the Ewaso N’giro River, the only permanent water in the area, and are a focal area for wildlife and tourism. Rainfall in the region averages approximately 350 mm per year (IQR: 242 mm to 401 mm) and occurs during biannual rainy seasons generally taking place in April/May and November/December. Due to the rainfall pattern in the system, demographic data were collated for the twelve month period between Oct. 1^st^ and Sept. 30^th^ in relation to the date of consistent separation between wet and dry periods in the ecosystem. Intermittent droughts (defined as years during which one of the wet seasons received no precipitation or when total annual rainfall was below the 25th quartile) affect the system periodically.

Individual identification using natural markings (protocols established in other populations [22], [23]) of all elephants of the reserves began in 1997 [16], since which time the elephants have been closely monitored. As a result of heavy tourist use of the parks, the reserve elephants are habituated to vehicles, enabling easy observation. The area used by these elephants is large (>3500 km^2^) and all elephants rely substantially on areas outside the protected areas [20]. Outside the protected areas, the elephants occur in largely unpatrolled, communally and government owned lands (with the exception of patrolled community conservation areas), where human elephant conflict and illegal killing occurs [24] and has significant impacts on the popualtion demography [21].

### Demographic Data

**Figure 2:**
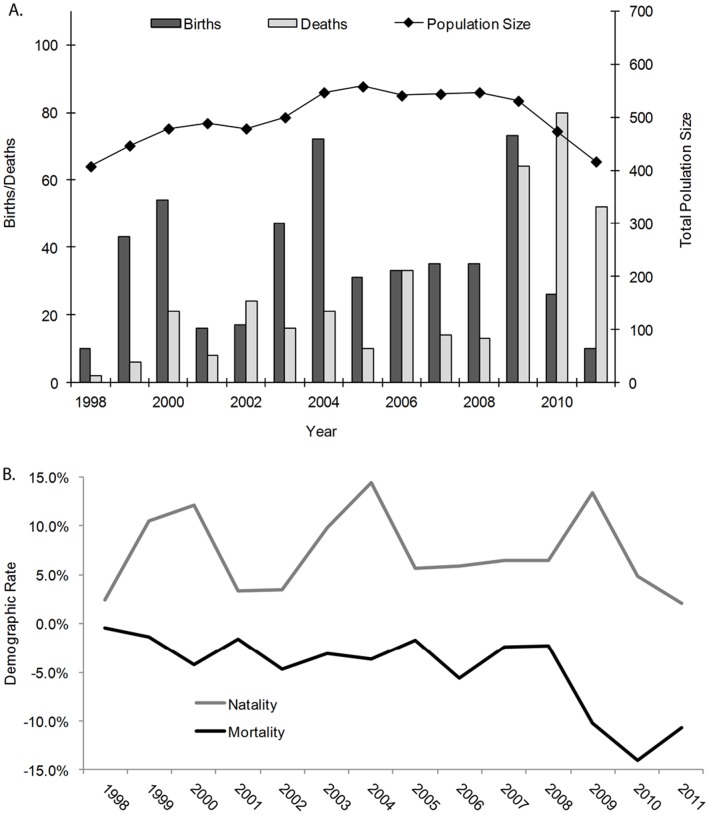
Annual Samburu population demography and population trend: (a) The number of births (black bars), deaths (gray bars), and the population size at the end of each year. (b) The population mortality and natality varied annually the duration of the study. Natality pulses every 3–4 years are a function of the prolonged (22 month) gestation period of elephant resulting in multi-year inter-calving intervals.

The data presented in this study were collected from November 1997 through September 2011 from resident elephants (as defined in [16]) in the national reserves, numbering between 408–558 individuals during the 14 year study (Fig. 2). Analysis was conducted on 509 individual females (accounting for 4628 live female years) and 425 males (accounting for 2914 live male years). The presence or absence of individual elephants, location, and time were recorded during weekly travel along 5 established transects (approximately 20 km long) in the protected areas [25], from which mortalities and births were deduced (see below). The study elephants are not always present in the national reserves [26], therefore sampling was opportunistic along these transects. During the 14 year study, 494 births and 340 deaths were recorded among these resident, focal elephants (Fig. 2) and 156 were thought to have dispersed (see description below). The average age at the first observation of new born elephants was 23 days (S.D. = 45). New adult females (a single family group) were identified in the population on one occasion in 2005, but were excluded from this analysis. New males periodically entered the population and when regularly observed were added to the studied cohort (n = 34).

Mortalities were identified in one of three ways as described in [19]: (1) known individuals were found dead; (2) repeated (>3) observations established individuals were missing from their family group; and (3) no observations for >3 years of individuals that previously had been observed annually by the monitoring team. None of the elephants recorded as dead were subsequently observed. Carcasses of the majority of registered mortalities were never found, therefore, the actual dates of death for these mortalities were unknown and year of death was assigned by adding its average inter-observation interval to the last date of observation as described in [19]. Carcasses were found during daily monitoring within the reserves (often initially sighted by tourists), but its identity was not always possible to assign depending on the age of the carcass, scavenger impacts, and cause of death (e.g. predation, disease, poaching). Researchers also investigated any reports of dead elephants in the wider ecosystem (approximately a 10 km buffer around the reserves) to assess if a carcass was a known individual. When the carcass of a known individual was found outside the reserves, relevant information (location, date and cause of death) were recorded and added to the standard monitoring data described previously. The cause of death of all carcasses found within the reserves and those of known individuals found outside the reserves were collated to derive the proportion of illegally killed elephants (PIKE = 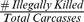) in the study area, the standard monitoring metric for the MIKE programme. In contrast to other demographic data presented here, PIKE figures were collated over the calendar year (Jan-Dec) to match continental protocol [17].

Because subadult males (3–18 years old) regularly leave their family groups and appear to range widely, they were recorded as dead only when their carcasses were observed or death could be inferred by other indicators (e.g. the presence of wounded family members). When subadult males transitioned from being integrated with the family, to trailing the family, to not being associated with the family (e.g. were no longer seen with their families but not known or suspected to have died), they were defined as dispersed and subsequently removed from the analyses (n = 156). As such, some individuals that died were likely treated as having dispersed. In the latter stages of the study when human induced mortality was high (2009–2011), some young females (orphans) from poached families were lost and could not be assumed dead since they were inconspicuous (e.g. indistinguishable from their age mates) and their regular associates were killed. These lost females were treated as dispersed, though it is likely some died. Due to these lost individuals, presented mortality levels should be considered minimums.

Of the 940 elephants in this study, the age of 596 individuals (63%) were known (i.e. they were observed within1.5 years of the estimated date of birth) with the rest estimated. Visual characteristics established from elephants of known age [27] were used to estimate the age of individuals and these age estimates were validated in the study population by comparing visual estimates of age with ages of dead or anesthetized individuals determined from dentition [28]. Age estimates of mature individuals based on physical appearance were within ±3 years of the age based on molar progression for 80% of the elephants [28].

### Analysis

Annual counts of live, dead and new born elephants were collated and used to derive population trends and time specific age structure in the population. Standard life table analyses were conducted to derive age specific mortality, life expectancy, reproductive number, and generation time [29]. The survival functions for the right censored and left truncated data on males and females were calculated as 
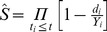
, where *d_i_* is the number of individuals that died at time *t_i_* and *Y_i_* is the number of individuals at risk at time *t_i_* [30]. Sex specific survival curves were derived for the low (1998–2008) and high (2009–2011) mortality periods, a division based on the year when the population began to decline. Survival functions were used to assess age specific reproductive onset among females (data on reproductive viability were not available for males) and age specific natal group dispersal among males (females do not disperse from their natal group). Dispersed individuals lost during the study (i.e. not observed post dispersal) were removed from successive analyses as of the last year of definitive identification. A log-rank test of Kaplan-Meier survivorship [30] was used to compare survival across sexes and between populations (derived from individual based monitoring: Addo Elephant Park [31], Amboseli National Park [27], [32], Tarangire National Park [33], [34]; data from culling: Kruger National Park [35], [36]) and hazard functions of reproductive onset between populations, as implemented in R 2.14.1 OIsurv library [37]. The compilation of demographic parameters derived for Samburu were only available for the Amboseli N. P. study, which was conducted using the same individual based approach [27], [32]. Therefore, comparison was focused on these two populations and included the other populations when possible. Only known age individuals, i.e. those identified when estimated to be 18 months or younger, were used in analysis of male dispersal and only females estimated to be under the age of 5 at identification were used in analysis of reproductive onset.

Analysis of demographic data from 12 different populations across Africa [22], [27], [31], [33], [36], [38], [39], [40], [41], [42] was conducted to assess the relationship between the response variables of age at first calving, inter-calving interval and annual mortality and covariate data on population density estimates and average annual rainfall in the study ecosystem (the only dependent and independent variables available across all populations). Information theoretic approaches (bias-adjusted Akaike’s information criterion (AIC_c_)) [43] were used to compare performance of generalized linear models with a Gaussian link function and results from the top model were presented.

## Results

This known sample of the elephant population of Samburu fluctuated from an initial 410 individuals at the beginning of 1998 to a peak of 558 in 2005 (the population was relatively stable between 2004–2008), and declining to 417 at the end of 2011 (Fig. 2a). Annual population growth (including the effects of immigration and emigration) averaged 0.17% over the fourteen year study, but was highly variable increasing during the early years of the study (with a maximum annual increase of 9.1% in 1999) and decreasing during the latter half of the study (with a maximum annual decline of 12.8% in 2011). Net change through migration (immigration plus emigration) averaged −1.4% (S.D. = 1.5%) per year, predominantly in the form of young male dispersal from their natal groups. Excluding migration, annual population growth averaged 2.9% (S.D. = 7.33%) per annum. Annual mortality varied broadly during the fourteen year study, averaging 4.71% (S.D. = 4.09%) per annum with a peak of 14.1% in 2010 at the end of a major drought spanning 2009–2010 (Fig. 2b). Prior to the onset of the major drought in 2009, mortality averaged 2.82% (S.D. = 1.57%) per annum. Similarly, annual natality was highly variable averaging 7.21% (S.D. = 4.10%) per annum, with a maximum of 14.4% in 2004 and a minimum of 2.1% in 2011 (Fig. 2b). The low recruitment in 1998 and 2011 (Fig. 1a) is the result of drought induced lows in fecundity [44].

**Figure 3:**
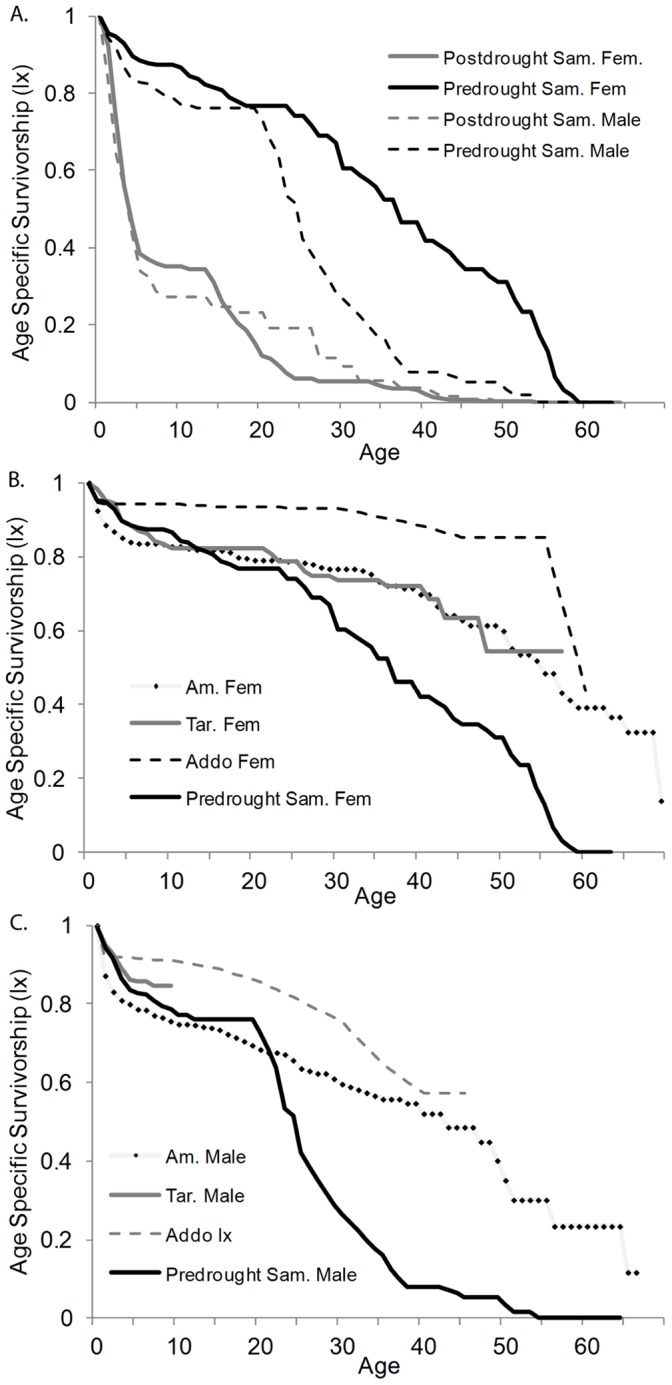
Comparative survivorship: (a) Survivorship curves for Samburu females (solid lines) and males (dashed lines) before (black) and after (gray) 2009 demonstrate the ramification of morality increases associated with the extreme drought of 2009 and successive increased illegal killing. (b) Survivorship among females in Samburu predrought (black line) was lower than that of females from Amboseli N. P. (gray line black dots), Tarangire N. P. (gray line), and Addo Elephant Park (dashed line). (c) Survivorship among males in Samburu predrought (black line) was lower than that of males from Amboseli N. P. (gray line black dots) and Addo Elephant Park (dashed line). Male survivorship was recorded only to the age of 8 years in Tarangire N.P. (gray line).

Female and male survival to the age of 10 years was 70% and 64% respectively over the fourteen year study, with the majority of deaths occurring between 2009–2011 during which survival to 10 years was estimated at 34% and 27% respectively (Fig. 3a; see Table S1). Male survival was significantly lower than female survival in Samburu over the fourteen year study (χ^2^ = 114, d.f. = 1, p<0.001; Fig. 3a). Age specific survival among the Samburu elephants was the lowest recorded in an intensively studied population, with Samburu female survival between 1998–2008 (mean = 22.3±1.59 years) significantly lower than the females of Amboseli (χ^2^ = 27.5, d.f. = 1, p<0.001) and Tarangire (χ^2^ = 9.3, d.f. = 1, p = 0.002) and Samburu male survival (mean = 19.3±1.35 years) during this period significantly lower than the males of Amboseli (χ^2^ = 872, d.f. = 1, p<0.001) (Fig. 3b (females) and 3c (males)). Maximum life span in Samburu for females and males was estimated at 64 and 54 years respectively, with life expectancy at birth estimated at 21.81 years for females (1998–2008 = 33.54 years; 2009–2011 = 8.01 years) and 18.85 years for males (1998–2008 = 22.96 years; 2009–2011 = 9.42 years) in contrast to female life expectancy of 40 years and male life expectancy of 24 years in Amboseli [27].

**Figure 4:**
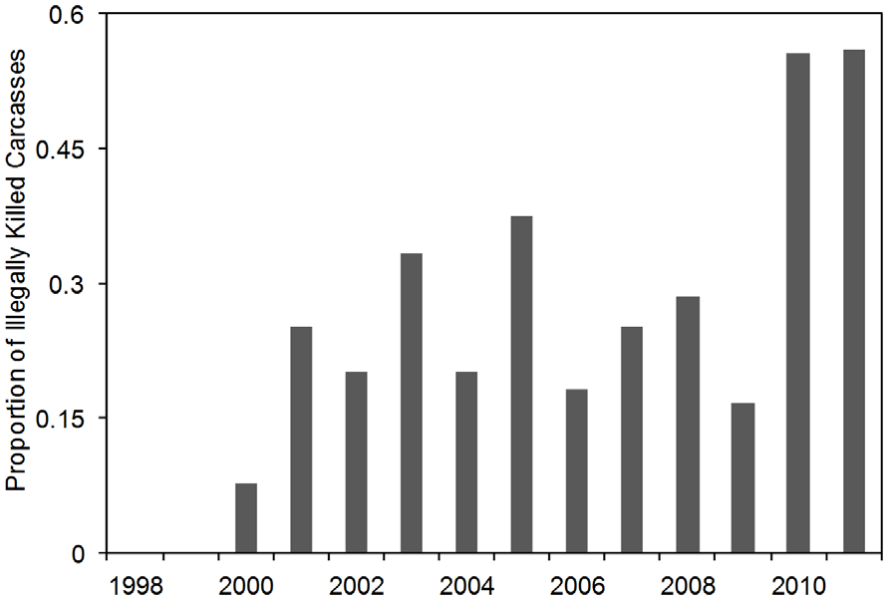
Annual proportions of illegally killed elephants (PIKE): The rate among found carcasses of illegally killing was greatest during the last three years of the study.

Illegal killing of elephants over the study period accounted for 32% of the known causes of death (i.e. those diagnosed from carcasses). Among elephants over the age of 9 years (approximate age of puberty), over half of the known deaths were from illegal killing (Table 1). The PIKE doubled in the last three years of the study, averaging 0.43 between 2009–2011 in contrast to 0.20 between 1998–2008, with the highest PIKE recorded in 2011 at 0.56 (Fig. 4).

**Table 1:** Causes of mortality in the Samburu population assessed from carcasses.

	Natural		Illegally Killed		Unknown		% Illegally Killed
Sex	M	F	M	F	M	F	
0–2	15	4	0	0	0	0	0%
3–9	17	13	2	2	4	1	10%
10–18	2	5	1	4	3	4	26%
19–30	6	3	3	10	1	1	54%
31–50	3	5	7	9	1	2	59%
50+	0	2	1	2	0	0	60%
Total	75		41		17		31%

**Figure 5:**
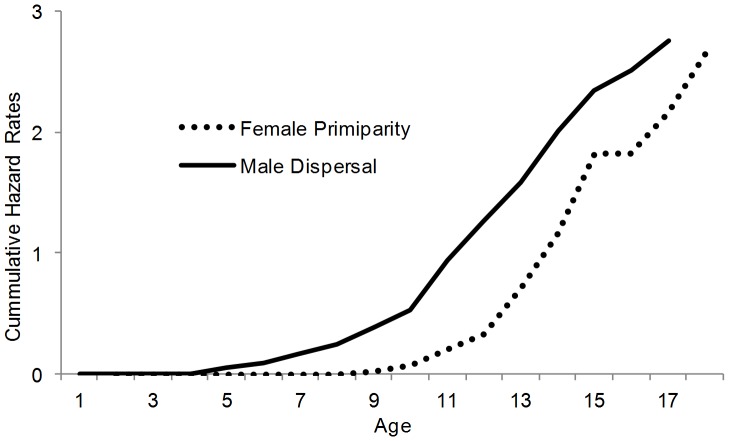
Indices of the age of reproductive onset: (a) Cumulative hazard rate for female primparous age (dotted line) and male dispersal (solid line) in the Samburu population.

**Figure 6:**
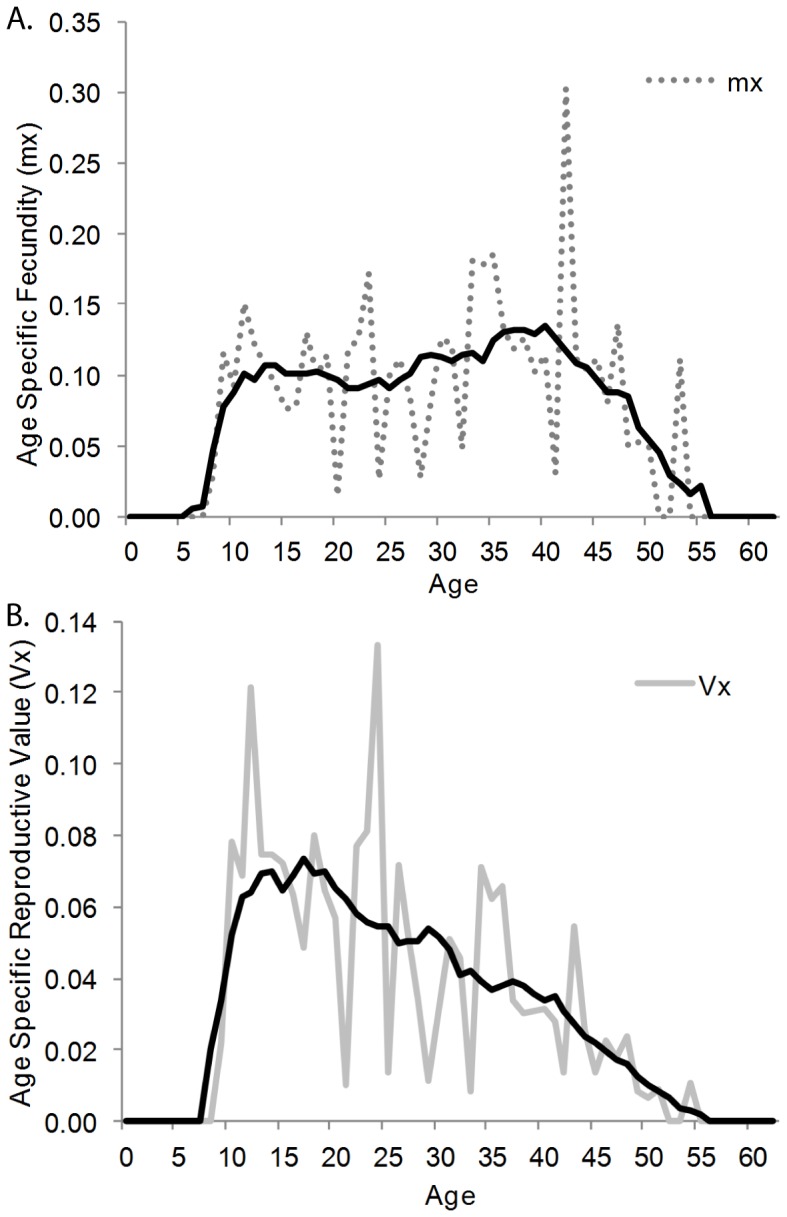
Summary of age class fecundity: (a) Age specific fecundity (m_x_) (dashed line = actual, and solid line = 5-year running average) peaked when the Samburu females were in their mid 40s. (b) Age specific reproductive value (v_x_) (dashed line = actual, and solid line = 5-year running average) declined after the peak between 15 and 20 years of age.

As calculated by standard life table approaches, the net reproductive number, R_0_, in the Samburu population was 1.59 and the mean generation time was 24.1 years. The youngest known primiparous age was 8.5 years and the oldest estimated age of first reproduction was 19 years. Analysis of primiparous females 8 years or older (n = 52) demonstrated the average age of primiparity in the population was 11.34 years (S.E. = 0.08) (Fig. 5). This was significantly lower than the average age of primiparity among Amboseli females (i.e. the Samburu mean was outside the Amboseli 95% C.I.) and historic populations with relatively high densities (Lake Manyara and Queen Elizabeth N.P.), and close to the ages of reproductive onset reported in the culled population of Kruger National Park and recovering Tarangire populations (Douglas-Hamilton 1972; Laws et al. 1975; Moss 2001; Foley and Faust 2010; Freeman et al. 2009). Age specific fecundity was 0.1 female calves per year (Fig. 6a), calculated as the number of female births per female year (female year is defined as the number of females alive at age x in the population). Fecundity was relatively constant between early and middle age, with age specific fecundity of 0.096 for females from 9–18 years (age span of primiparous females) and 0.092 for females 19–30 years. Fecundity was slightly higher for mature females between 30 and 50 years at 0.13 female calves per year, as a result of an increase in fecundity between 30 and 40 years of age. Similar to Amboseli N.P. [27], fecundity decreased after the age of 40 years. Only three of 20 females were observed to give birth in their 50’s with age specific fecundity at 0.032 females per year between 50 and 60 years. No female was observed to give birth after the estimated age of 54 years (Fig. 6a). Reproductive value declined linearly after its peak at age 10 years (Fig. 6b). Inter-calf intervals among females with calves identified within 30 days of birth (n = 264) averaged 4.01 years (S.D. = 0.94). This interval was reduced by approximately one year when a female lost her previous calf within the first year (n = 14), as found in Amboseli N.P. (Moss 2001).

Reproductive success of all known males was not possible to assess, but previous work indicates fecundity is skewed towards older age classes [45]. The youngest known male to disperse from his natal family unit was 5 years old and the oldest was 18 years old (Fig. 5). Analysis of male dispersal age (n = 111) in the population demonstrated a mean dispersal age of 9.66 years (S.E. = 0.07). Approximately one fifth (21%) of these dispersals occurred after the death of the male juveniles mother. Males as young as 5 years were observed to disperse from their natal group without any obvious disruptive inducement (e.g. mother death).

The number of elephants over the age of 30 years in the known population declined over the course of the study. At its peak in 2000, 38 known males were estimated to be over the age of 30 years, but by the end of 2011 only 12 constituted this age class (7 of which were recruited into the age class after 2000, i.e. only 5 of the original 38 survived to 2011). Similarly, known females over the age of 30 peaked in 2002 and 2006 at 59 individuals, but numbers were nearly halved between 2009 and 2011 with 32 remaining at the end of 2011 (Fig. S1). While some of this mortality was due to the serious 2009 drought, at least half is thought to have been caused by illegal killing (Table 1). This strong mortality altered the age structure and age-related social organization. The known population sex ratio has grown more skewed across the years with significantly more females than males across six age classes in 1998 (χ^2^ = 80.38, d.f. = 5, p<0.001) when 42% of the elephants were male, 2004 (χ^2^ = 71.81, d.f. = 5, p<0.001) when 41.6% were male, and 2011 (χ^2^ = 335.51, d.f. = 5, p<0.001) when 32% were male (Fig. S2). Social groups as defined in [25] have also changed dramatically, with 10 of the 50 quantitatively defined groups being extirpated (no known breeding females surviving) and 13 with no breeding female over the age of 25 years (Fig. S3).

The best supported model in an analysis of primiparous age (average = 13.4 years) across 12 populations (Table 2) demonstrated a significant positive correlation with the interaction term rainfall and density (*t* = 2.80, *p* = 0.021). The best supported model of inter-calving interval (average = 5 years) demonstrated a positive relationship with rainfall (*t* = 2.871, *p* = 0.015), excluding density (*t* = −0.197, *p* = 0.847) as a predictive variable. The best supported model of mortality (average = 4.1% per year across the 11 populations with reported values) showed a non-significant negative relationship with density (*t* = −0.708, *p* = 0.497).

**Table 2:** African elephant demographic parameters from 12 wild populations (one sampled twice) collected through individual registration, radio tracking or culling.

	YoungestConception Age	Avg. PrimiparousAge (years)	Mean CalvingInterval (years)	AnnualMortality	Densityele/mile^2^	Yearly Rain(mm)	Citation
Addo N.P. South Africa	9	12.5	3.3	1.4%	9.6	392	Gough and Kerley 2006
Amboseli N.P. Kenya	7.1	13.7	4.5	4.15%	<1	350	Lee and Moss pers comms. 2012, Moss 2001
Etosha N.P. Namibia	–	11.1	3.8	5.0%	<1	250–450	Lindeque 1988
Kruger N.P. South Africa	8	13	3.8	3.2%	<1	450–700	Whyte et al. 1998, Freeman et al. 2009
Lake Manyara N.P. Tanzania	8	13	4.6	3.5%(2.7–3.9%)	6.2	650	Douglas-Hamilton 1972
Luangwa N.P. Zambia	12	16	3.4	–	3–6	800	Hanks 1972
Mkomazi N.P. Tanzania	–	12.2	4.2	5.0%	2.5	400	Laws et al. 1975
Murchison Falls N.P. Uganda	7	11	8.6	–	1.7	1,085	Buss and Smith 1966
Murchison North Uganda	–	16.3	9.1	5.1–6.6%	3–4	1,085	Laws et al. 1975
Murchison South Uganda	–	17.8	5.6		6–7	1,085	Laws et al. 1975
Samburu N.R. Kenya	6.7	11.5	4.0	2.8% pre4.7% total	<1	350	This study
Tarangire N.P. Tanzania	6.9	11.2	3.3	1.9%	<1	656	Foley and Faust 2010
Tsavo N.P. Kenya	–	14.5	6.8	3.8–5.0%	3	200–700	Laws et al. 1975
Captive	12	16.3	–	–			Dale 2010

## Discussion

In addition to providing detailed population level demographic data, the Samburu study offers the first individually based demographic analysis of the impacts of illegal killing, offering detailed insight to demographic parameters (survival, fecundity, and population level metrics) in the face of moderate to high human pressures. Such pressure increased as the study progressed, allowing contrasts between periods of increase/stability and decline in a single population. Unfortunately, illegal killing and related population decline is increasingly common across Africa [17], [18], therefore the results from this study are directly relevant to understanding the conservation status of the species.

### Demographic Ramifications of Illegal Killing and Drought Induced Mortality

While survivorship was particularly low during the latter three years of the study, a period marked by relatively high illegal killing and intense drought, survivorship was significantly lower than the less impacted population of Amboseli even during the initial period (1998–2008) of increase/stability (Fig. 3b and 3c). Over the fourteen year study, life expectancy in Samburu among females was approximately half and males approximately a quarter of the less impacted population in Amboseli N. P. As predicted by life history theory [46], [47], reproductive effort was greater (earlier age of primiparity and shorter inter-calf interval) in the Samburu relative to the Amboseli population. These metrics in the Kruger elephant population, where high levels of mortality were induced by management based culling, were similar to those found in Samburu [35], [36], indicating savanna elephants may demonstrate a compensatory reproductive response to lower survival across systems. A similar response was proposed as the cause for the high reproductive surge in the Tarangire population, recorded during a period of recovery after extensive poaching impacted the population [33], [34], though survival during the period of data collection was similar to that in Amboseli (Fig. 3b). This may indicate a lag in the purported compensatory reproductive response relative to survival rates, potentially associated with changes in the age structure of the population (see below).

Below 25^th^ percentile rainfall (drought) years repeatedly caused declines in the survival of the younger age classes (see mortality in 2000, 2006 and 2010). During the acute drought of 2009–2010, survival of mature individuals declined as a result of both natural drought and a surge in illegal killing (with life expectancy dropping approximately 20 years during the latter portion of the study). The reduction of adult survival being partially a function of drought was unexpected given the drought resistant biology of mega-herbivores [48] and results from previous analyses from this population showing that ecological conditions poorly predicted adult mortality [19]. The impact of drought induced mortality was compounded by the related but lagged slowing of reproduction (as demonstrated in [44], [49]), which resulted in low recruitment in 2011 (10 total births) because of the long (22 month) gestation period of elephants. Therefore the impact of droughts on population growth was typically carried out over 2–3 years. The regulatory importance of natural ecological fluctuations to this species has been discussed in relation to management concerns [50], but the strength of the impact of a single year major drought (killing close to a sixth of the population and impacting the population for multiple years) may not have been recognized (though see [42], [51]).

Illegal human killing caused over half the recorded mortality in the Samburu elephants over the age of 9 (and indirectly caused the deaths of all victim’s dependent calves under 2 years). The high illegal killing in the latter part of the study had serious ramifications for the structure and organization of the population. In contrast to representative age structure culling [36], the illegal killing appeared to select adult individuals in Samburu and particularly males resulting in increasing skew in the sex ratio over the course of the fourteen year study (Fig. S1). Social disruption also resulted, with numerous well known and stable family groups being completely lost (i.e. no surviving breeding females) causing increased numbers of unaffiliated juveniles (orphans) [21]. These orphans take different strategies in relation to their social context, remaining solitary, clustering with other orphans or joining other groups. Such processes were first noted by genetic studies of the population [52], but the number of orphaned individuals has risen sharply. In addition, the relative density of individuals in older age classes has declined compared with densities during the study onset. It is critical to follow this population in the future to understand if such alterations impact demographic processes.

### Comparative Demography Across Elephant Populations

Demographic data on African elephants have been compiled from a variety of ecological settings from semi-arid to highly mesic savannas and span a gradient from high to low densities, offering an opportunity to investigate environmental correlates with different population parameters (Table 2). As identified by [41], higher densities generally were associated with slower reproduction (i.e. greater age of reproductive onset), though calving interval showed no relationship to density among the populations assessed. Annual mortality was not correlated with either density or rainfall. Most demographic data available from high density populations happen to be located in mesic environments, however, reducing the ability to draw inference on the interaction between density and ecological conditions at this scale of analysis. The interaction between density and fecundity was not linearly predictable, with Lake Manyara N.P. [22] demonstrating relatively fast reproduction among the high density populations though slower reproduction than the low density, human impacted populations. The slower reproduction in Amboseli relative to Samburu, Tarangire, and Kruger was not attributable to density (with all at relatively low densities); an older age structure in Amboseli may be the most significant difference [27], but how this influences fecundity remains unclear. Evidence of density dependence was not found in demographic studies of the highly managed Addo system [31], a population which periodically reached high densities, but for which the impacts of density were mitigated through park expansion and resource provisioning that dampened ecological processes that influence elephant populations [53]. Within the Samburu population, inter annual fluctuations in survival and fecundity associated with rainfall can be interpreted as resulting from density dependent effects, with high mortality induced by droughts (lower carrying capacity) and birth pulses resulting from mesic years (high carrying capacity).

### Management Implications of Samburu Demography

The contrast between the conservation status of Amboseli and Samburu despite broadly similar management contexts is particularly salient to assessment of this species’ status. Both populations inhabit similar semi-arid ecosystems (average annual rainfall in both is ∼350 mm) and are located in Kenyan protected areas that cover a small proportion of their home ranges, for which security is maintained by the Kenya Wildlife Service (though Amboseli is a national park in contrast to the national reserve status in Samburu) [24], [27]. Rather than ecological or protective institutional differences, the differences in human pressure across these populations is related to the local political context and instability in the ecosystems, with the diverse ethnic landscape in Samburu being prone to more frequent unrest relative to the homogeneous ethnic context around Amboseli [24], [32]. The critical ramifications of local political and economic contexts on population conservation status [19] highlights potential weaknesses in analyses focused predominantly on national scale indicators to understand human drivers of demographic processes [17].

Finally, basic life table metrics derived from the Samburu data provide information required for red list assessments as specified by the IUCN [54]. One of the key demographic parameters applied in red list assessments is generation time, on which the period considered (3x the generation time) when assessing changes in numbers or distribution are based [55]. The generation time in Samburu (24.1 years) was approximately equal to the 25 years calculated for Kruger, but the combined decreased survivorship and increased fecundity (early age of primiparity and shorter inter-calving interval) found in these human impacted populations potentially decrease generation time relative to that of an unperturbed population. The 17.4 year generation time reported for Amboseli was calculated using both sexes [27], not strictly females. Therefore, the standard generation time for Amboseli would be higher. Despite the faster reproduction, age specific fecundity and reproductive value were broadly similar to those reported for Amboseli (Amboseli values included both males and females in their assessment, but due to their reported 1∶1 sex ratio can be halved for comparison), with reproductive output peaking in mid-aged females and declining in older age classes indicating senescence [56].

These data from Samburu and their comparison to other populations provide a more comprehensive understanding of the demographic processes occurring across populations in this species. As such, these data can facilitate more accurate modeling of elephant population dynamics across the spectrum of conditions they encounter. In modeling approaches, however, it is important to recognize site specific factors structure demographic processes, limiting inference provided by extrapolating from weak correlations between demographic parameters and coarse ecological indices.

## Supporting Information

Figure S1 **Change in number of older age class individuals: The number of mature adult (30 years or older) males (gray line) declined consistently between 2000 and 2011, while mature females (black line) declined rapidly during the last three years of the study.**
(TIF)

Figure S2 **Change in population sex ratio: The sex ratio among the closely monitored elephants has increasingly become skewed with fewer males.**
(TIF)

Figure S3 **Change in population social structure: High mortality in the latter half of the study caused severe social disruption for nearly half of the intensively monitored social units.** Disrupted groups had no breeding females over the age of 25 years and extirpated groups had no remaining breeding females during the specified year.
(TIF)

Table S1 **Female and male age specific survivorship.**
(DOCX)
