# Supplementary figures and images for: Comparative Demography of an At-Risk African Elephant Population
Source: PLoS One. 2013 Jan 16;8(1):e53726. doi: 10.1371/journal.pone.0053726 (PMC3547063; doi:10.1371/journal.pone.0053726)

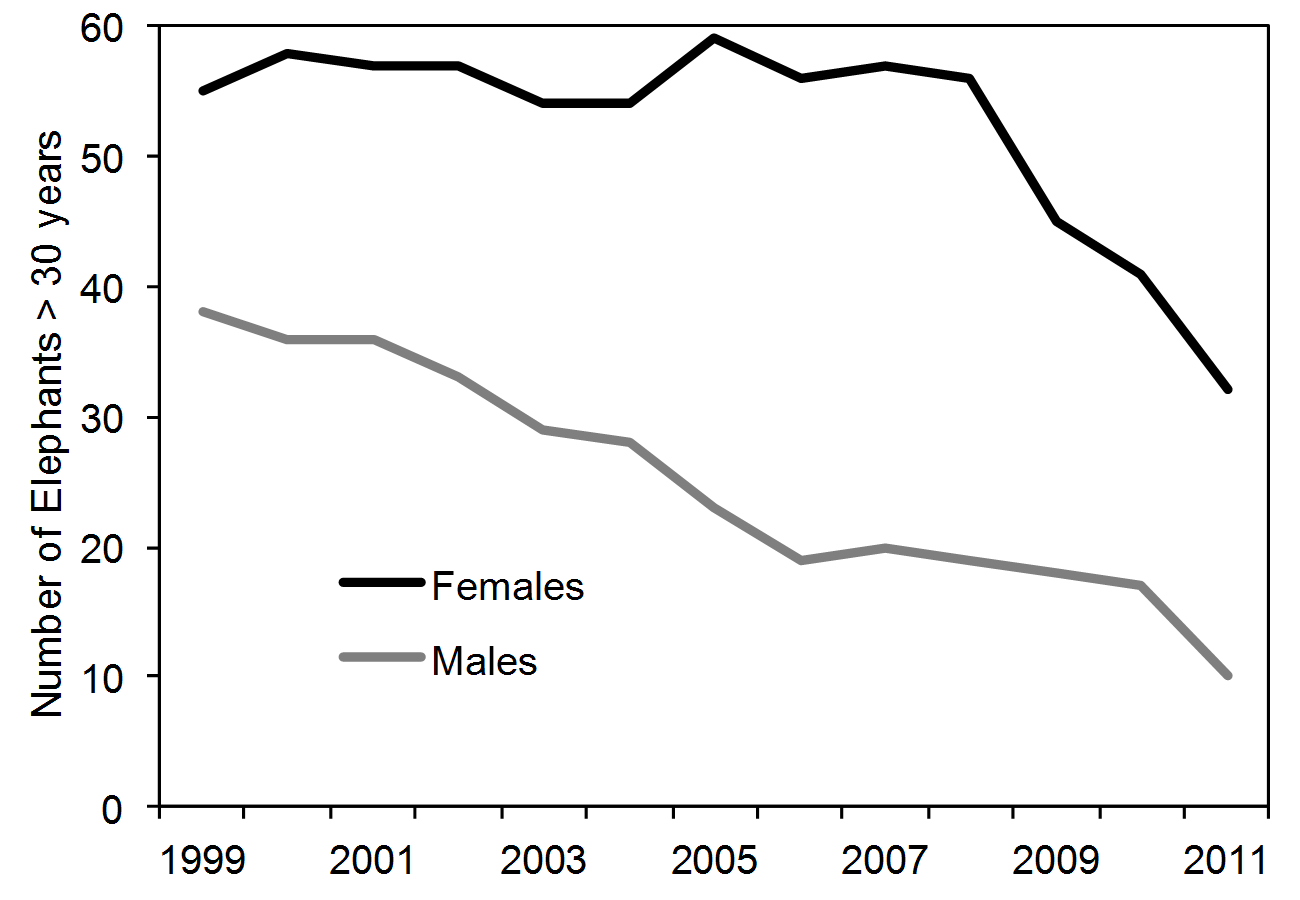

Supplement: Figure S1 — Change in number of older age class individuals: The number of mature adult (30 years or older) males (gray line) declined consistently between 2000 and 2011, while mature females (black line) declined rapidly during the last three years of the study. (TIF) [file pone.0053726.s001.tif]

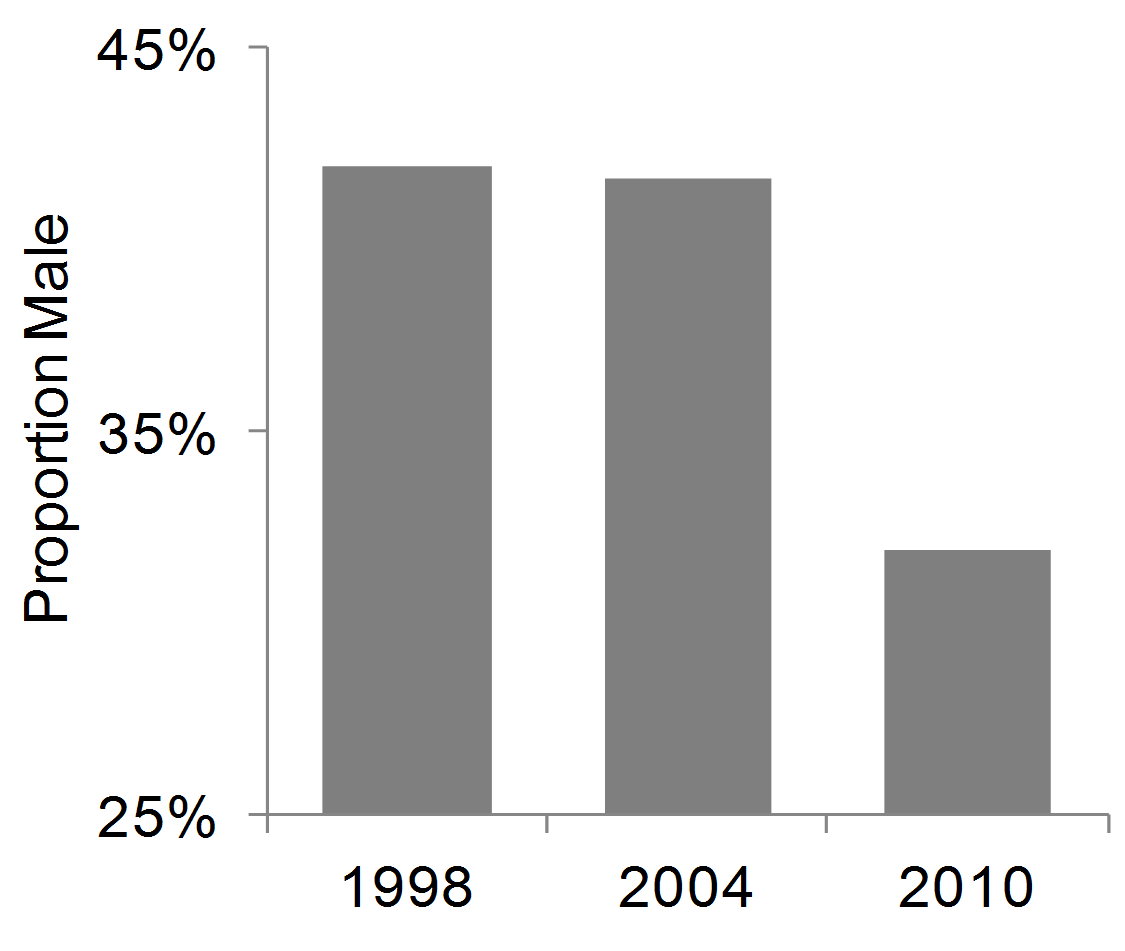

Supplement: Figure S2 — Change in population sex ratio: The sex ratio among the closely monitored elephants has increasingly become skewed with fewer males. (TIF) [file pone.0053726.s002.tif]

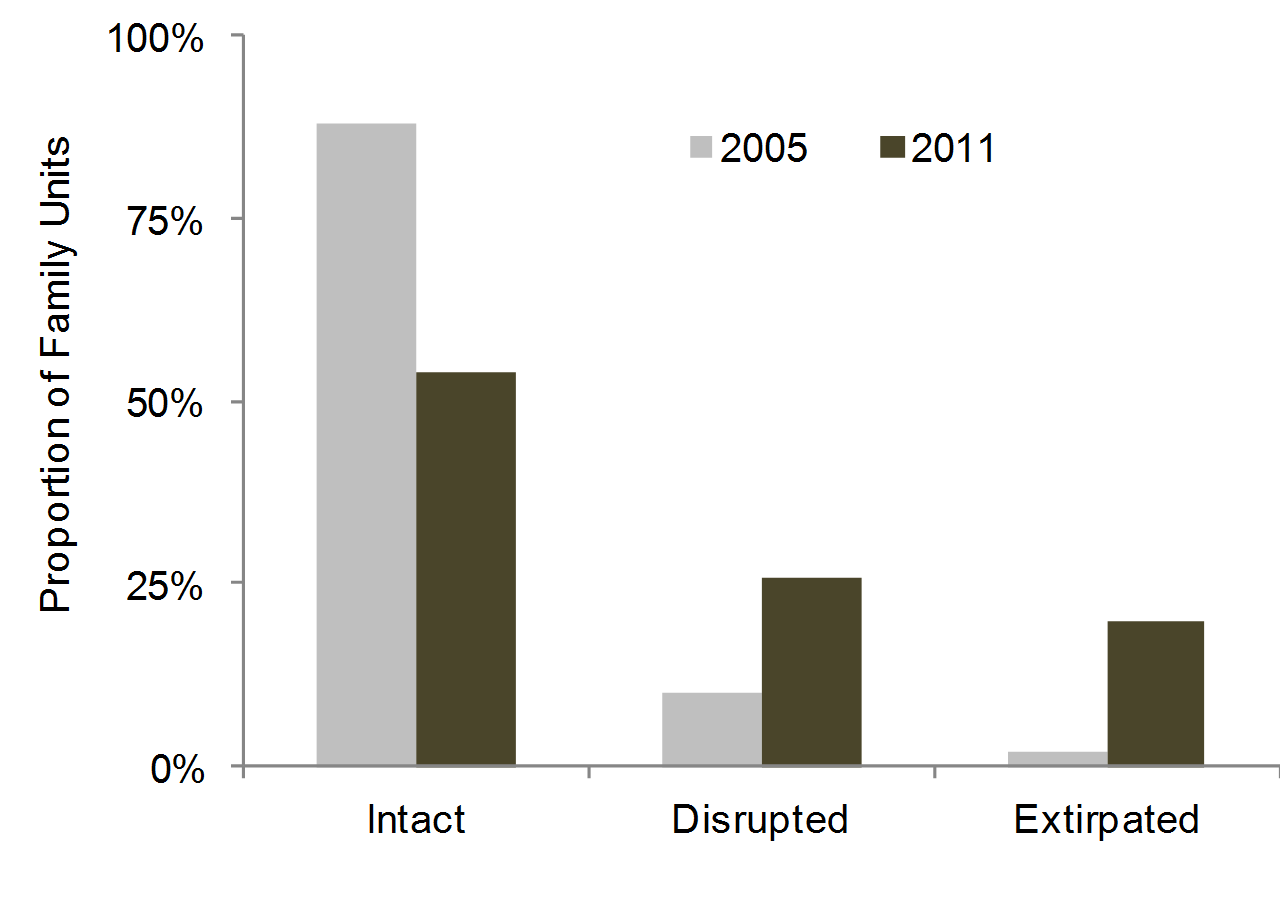

Supplement: Figure S3 — Change in population social structure: High mortality in the latter half of the study caused severe social disruption for nearly half of the intensively monitored social units. Disrupted groups had no breeding females over the age of 25 years and extirpated groups had no remaining breeding females during the specified year. (TIF) [file pone.0053726.s003.tif]
