# Supplementary material for: Comparative Demography of an At-Risk African Elephant Population
Source: PLoS One. 2013 Jan 16;8(1):e53726. doi: 10.1371/journal.pone.0053726 (PMC3547063; doi:10.1371/journal.pone.0053726)
Supplement: Table S1 — Female and male age specific survivorship. (DOCX) [file pone.0053726.s004.docx]

**Supporting Information**

*Comparative demography of an at-risk African elephant population*

George Wittemyer, David Daballen, Iain Douglas-Hamilton

Correspondence should be addressed to G.W. (G.Wittemyer@ColoState.edu)

Supplemental Table

Table S1: Female and male age specific survivorship

| *Age* | *Female* | *Male* |
| --- | --- | --- |
| 0 | 1.000 | 1.000 |
| 1 | 0.946 | 0.924 |
| 2 | 0.892 | 0.855 |
| 3 | 0.815 | 0.795 |
| 4 | 0.762 | 0.747 |
| 5 | 0.729 | 0.714 |
| 6 | 0.717 | 0.701 |
| 7 | 0.706 | 0.670 |
| 8 | 0.703 | 0.652 |
| 9 | 0.703 | 0.647 |
| 10 | 0.698 | 0.635 |
| 11 | 0.681 | 0.635 |
| 12 | 0.677 | 0.628 |
| 13 | 0.667 | 0.628 |
| 14 | 0.652 | 0.617 |
| 15 | 0.616 | 0.617 |
| 16 | 0.590 | 0.617 |
| 17 | 0.567 | 0.595 |
| 18 | 0.549 | 0.595 |
| 19 | 0.513 | 0.595 |
| 20 | 0.467 | 0.573 |
| 21 | 0.454 | 0.528 |
| 22 | 0.424 | 0.499 |
| 23 | 0.393 | 0.421 |
| 24 | 0.356 | 0.408 |
| 25 | 0.356 | 0.342 |
| 26 | 0.347 | 0.316 |
| 27 | 0.327 | 0.272 |
| 28 | 0.327 | 0.247 |
| 29 | 0.319 | 0.220 |
| 30 | 0.293 | 0.200 |
| 31 | 0.293 | 0.184 |
| 32 | 0.286 | 0.162 |
| 33 | 0.279 | 0.145 |
| 34 | 0.266 | 0.127 |
| 35 | 0.248 | 0.118 |
| 36 | 0.243 | 0.094 |
| 37 | 0.217 | 0.073 |
| 38 | 0.217 | 0.060 |
| 39 | 0.212 | 0.060 |
| 40 | 0.179 | 0.060 |
| 41 | 0.169 | 0.057 |
| 42 | 0.159 | 0.049 |
| 43 | 0.149 | 0.044 |
| 44 | 0.132 | 0.040 |
| 45 | 0.119 | 0.030 |
| 46 | 0.119 | 0.030 |
| 47 | 0.109 | 0.030 |
| 48 | 0.104 | 0.030 |
| 49 | 0.099 | 0.026 |
| 50 | 0.099 | 0.015 |
| 51 | 0.084 | 0.011 |
| 52 | 0.058 | 0.007 |
| 53 | 0.047 | 0.007 |
| 54 | 0.042 | 0.000 |
| 55 | 0.030 | 0.000 |
| 56 | 0.030 | 0.000 |
| 57 | 0.012 | 0.000 |
| 58 | 0.012 | 0.000 |
| 59 | 0.012 | 0.000 |
| 60 | 0.012 | 0.000 |
| 61 | 0.012 | 0.000 |
| 62 | 0.012 | 0.000 |
| 63 | 0.000 | 0.000 |
